# Interstitial Lung Disease: Comparing Lecture and Large Group Case-Based Learning for Preclinical Medical Students

**DOI:** 10.7759/cureus.63100

**Published:** 2024-06-25

**Authors:** Juliana Cazzaniga, Matthew Shatanof, Gagani Athauda, Jenny Fortun, Amilcar Castellano, Juan Lozano, Rebecca Toonkel

**Affiliations:** 1 Medicine, Florida International University, Herbert Wertheim College of Medicine, Miami, USA; 2 Research, Florida International University, Herbert Wertheim College of Medicine, Miami, USA; 3 Cellular Biology and Pharmacology, Florida International University, Herbert Wertheim College of Medicine, Miami, USA; 4 Pathology, Florida International University, Herbert Wertheim College of Medicine, Miami, USA; 5 Translational Medicine, Florida International University, Herbert Wertheim College of Medicine, Miami, USA; 6 Internal Medicine, Florida International University, Herbert Wertheim College of Medicine, Miami, USA

**Keywords:** diagnostic testing, case based learning, active teaching-learning, preclinical medical students, progressive interstitial lung disease

## Abstract

Introduction and objective

Interstitial lung disease (ILD) is a subject with which preclinical medical students often struggle. Because case-based learning (CBL) engages students in discussions centered around complex clinical scenarios, it may be effective for teaching ILD to preclinical medical students by fostering the development of critical thinking and clinical reasoning skills.

Methods

Lecture-based learning on ILD in the second-year Respiratory System course was replaced with a large group CBL session. Students worked in a large group team-based CBL (TB-CBL) to answer questions about the etiology, presentation, diagnostic radiology images, pathophysiology, and management of various ILDs. Performance on ILD-related final exam questions from Cohort A (Class of 2023, lecture) was compared with Cohort B (Class of 2025, TB-CBL). Student satisfaction was assessed through an anonymous end-of-course survey (5-point Likert).

Results

Mean performance on ILD-related final exam questions was 85.4 (SD: 16.5) for Cohort B (TB-CBL) vs. 80.0 (SD: 17.6) for Cohort A (lecture). Mean overall satisfaction was 3.31 (SD: 1.37), with 87.6%, 88.4%, and 87.6% agreeing or strongly agreeing with the statements “The session was well organized,” “The session contributed to my learning,” and “The session was a valuable use of my time,” respectively.

Conclusion

Students participating in TB-CBL scored higher on ILD-related final exam questions compared with those who received lecture-based learning of the same material. Student satisfaction was acceptable but lower than expected. This TB-CBL session may be adapted at other institutions aiming to utilize active learning methods to teach the principles of diagnosis and management of ILD to preclinical medical students.

## Introduction

Interstitial lung disease (ILD) refers to a complex and heterogeneous group of over 200 disorders. ILD represents a diverse group of disorders characterized by inflammation and fibrosis of the lung parenchyma, leading to impaired gas exchange and progressive respiratory failure. The term encompasses a broad spectrum of conditions, including idiopathic pulmonary fibrosis (IPF), sarcoidosis, connective tissue disease-associated ILD, hypersensitivity pneumonitis, and others. ILD poses a significant clinical challenge due to its heterogeneous nature, varied etiology, and often insidious onset [1].

Because delays in the diagnosis of ILD may result in lower survival rates and higher healthcare costs [2,3], it is crucial for medical students to be well trained in identifying, diagnosing, and treating ILD. Accordingly, these topics are included in the United States Medical Licensing Exam Content Outline [4]. The heterogeneity in clinical presentation and pathogenesis of the various ILDs makes it challenging for medical students to master this topic without clinical context. Thus, this topic may best be taught through active learning modalities.

Evidence demonstrates that adopting more active learning techniques is linked to better student learning outcomes [5,6]. Active learning modalities such as case-based learning (CBL) incorporate clinical reasoning and application of knowledge [7], with abundant literature documenting the success of CBL across a broad range of topics in medical education [7-14].

Similar to CBL, large group team-based CBL (TB-CBL) incorporates the teamwork aspect of team-based learning while simultaneously upholding the structural and clinical reasoning elements of the CBL paradigm [9]. The TB-CBL format has been shown to be effective while also minimizing cost and heterogeneity in the learning experience compared with traditional small-group CBL [10].

While CBL is commonly used in undergraduate medical education [13-15], to our knowledge, such an approach has not yet been applied to ILDs. While the Pulmonary Fibrosis Foundation offers continuing medical education resources for providers [16], our search of the literature, including Google Scholar and PubMed, reveals no active learning resources outside of the MedEdPORTAL database, which itself only contains three relevant publications on general lung diseases, not ILD specifically [17-19].

To fill this need, we developed and implemented a TB-CBL session consisting of five short cases designed for preclinical medical students. Through this session, students apply the principles of diagnosis, testing, and treatment of IPF, sarcoidosis, hypersensitivity pneumonitis, nonspecific interstitial pneumonitis, drug-related parenchymal lung disease, and pneumoconioses, including silicosis, asbestosis, and coal worker’s lung. This session is designed to incorporate material across courses and disciplines in an active learning format. In contrast with other published reports, our session focused directly on ILD, from risk factors and presentation through diagnosis and treatment.

## Materials and methods

Session design and implementation

Prior to April 2021, students at the Florida International University, Herbert Wertheim College of Medicine, Miami, USA, were taught ILD through a 90-minute clinical lecture and a one-hour pathology lecture during the second-year Cardiovascular and Respiratory Systems course. After April 2021, students participated in a new TB-CBL session designed by a team of basic science and clinical faculty with expertise in pulmonary disease and co-facilitated by a pulmonologist and a pathologist. In order to minimize confounders, this study collected data from Cohort A (students in the Class of 2023), who covered ILD in a lecture format before the COVID-19 pandemic, and Cohort B (students in the Class of 2025), who participated in the in-person TB-CBL session. This approach excluded analysis of the virtual sessions conducted via Zoom during the pandemic.

Prior to participating in the TB-CBL session, students were required to review a pre-session study guide detailing ILD classification, pathophysiology, signs, and symptoms, and student readiness was assessed through a pre-session readiness quiz consisting of eight multiple-choice questions (MCQs).

During the three-hour session, a PowerPoint presentation detailing the cases and specific questions was projected to the large group. Students were randomly assigned to numbered groups of three and instructed to work together to document their answers. For each question, facilitators provided students with time to discuss independently and then used a bingo ball roller to randomly select one group to present their answer to the larger class. Facilitators then guided the larger group discussion by asking probing questions, summarizing, managing time, and maintaining order.

Outcome measures and evaluation

Knowledge gains were measured by performance on ILD-related MCQs included on the course final exam administered to both Cohort A (Class of 2023, lecture, n = 129 students) and Cohort B (Class of 2025, TB-CBL, n = 139 students). Additional analysis was performed to identify potential differences in performance between the cohorts on questions defined as recognition style (requiring simple recall of information presented in class) vs. application style (requiring the use of information presented in class to solve a clinical question). Overall final exam performance (n = 71 for Cohort A and n = 68 for Cohort B) was compared to assess potential differences in baseline cohort performance.

Student satisfaction with the TB-CBL session was assessed through three questions (Likert 1-5, strongly disagree through strongly agree) and an open comment section on the anonymous end-of-course survey administered to Cohort B.

An independent two-sample t-test was used to compare knowledge acquisition between lecture-based teaching (Cohort A) and TB-CBL (Cohort B). Satisfaction was calculated using the mean statement agreement. Thematic analysis of open comment responses was completed to identify potential patterns. All calculations were performed using IBM SPSS Statistics for Windows, Version 26.0 (Released 2019; IBM Corp., Armonk, NY, USA).

This study received IRB-exempt approval (#IRB-108639) at Florida International University.

## Results

Mean performance on ILD-related final exam questions was 85.4 (SD: 16.5) for Cohort B (TB-CBL) vs. 80.0 (SD: 17.6) for Cohort A (lecture), resulting in a mean difference of 5.4 (95% CI: 1.3-9.5, p = 0.01, Table 1). A difference was seen in mean performance on recognition-style questions (92.8 (SD: 23.8) for Cohort B vs. 84.5 (SD: 30.3) for Cohort A, p = 0.013); however, no difference was found in mean performance on application-style questions (81.7 (SD: 38.4) for Cohort B vs. 77.7 (SD: 39.1) for Cohort A), p = 0.399). Of note, a significant difference was found in overall final exam performance between the cohorts (87.3 (SD: 4.7) for Cohort B vs. 85.3 (SD: 4.5) for Cohort A, p = 0.001).

**Table 1:** ILD-related final exam performance ILD, interstitial lung disease; TB-CBL, team-based case-based learning

Question(s)	TB-CBL mean (SD)	Lecture mean (SD)	Mean difference (95% CI)	p-value	Question Style
1	74.1 (44.0)	56.6 (49.8)	17.5 (6.2 to 28.8)	0.003	Application
2	86.3 (34.5)	81.4 (39.1)	4.9 (-4.0 to 13.8)	0.275	Application
3	97.8 (14.6)	97.7 (15.1)	0.2 (-3.4 to 3.8)	0.927	Recognition
4	84.2 (36.6)	82.2 (38.4)	2.0 (-7.0 to 11.0)	0.663	Application
5	87.8 (32.9)	71.3 (45.4)	16.5 (6.9 to 26.1)	0.001	Recognition
6	82.0 (38.6)	90.7 (29.2)	-8.7 (-16.9 to -0.5)	0.038	Application
Application only	81.7 (38.4)	77.7 (39.1)	-4.0 (-13.3 to 5.3)	0.399	Application
Recognition only	92.8 (23.8)	84.5 (30.3)	-8.3 (-14.8 to -1.8)	0.013	Recognition
All ILD questions	85.4 (16.5)	80.0 (17.6)	5.4 (1.3 to 9.5)	0.01	Both
Overall exam performance	87.3 (4.7)	85.3 (4.5)	-2.0 (-3.1 to -0.9)	0.001	Both

As shown in Table 2, 82% (n = 114 of 139) of students in Cohort B completed the anonymous end-of-course survey. Mean overall satisfaction was 3.31 (SD: 1.37), with 87.6%, 88.4%, and 87.6% agreeing or strongly agreeing with the statements “The session was well organized,” “The session contributed to my learning,” and “The session was a valuable use of my time,” respectively.

**Table 2:** ILD TB-CBL satisfaction survey results ^a ^5-point scale (1 = strongly disagree; 5 = strongly agree) ILD, interstitial lung disease; TB-CBL, team-based case-based learning

Statement	Mean^a^ (SD)	% Agree or strongly agree
“Session was well organized”	3.29 (1.35)	87.6
“Session contributed to my learning”	3.30 (1.39)	88.4
“Session was a worthwhile use of my time”	3.35 (1.37)	87.6
Overall	3.31 (1.37)	87.9

A qualitative review of open comments (Table 3) revealed that several students noted the session’s contribution to learning. with a few recognizing its value toward consolidation and scaffolding. While several students expressed general satisfaction, many others expressed sentiments favoring lecture over TB-CBL. Areas for improvement identified included the thoroughness of the preparatory material, its alignment with the session content, and the organization and scope of the in-class session.

**Table 3:** Qualitative themes identified from student comments ILD, interstitial lung disease; TB-CBL, team-based case-based learning

Category	Number	Representative comments
Strengths (n = 11)		
General contribution to learning	3	“This session contributed to my learning on ILD.”
General satisfaction	6	“Good session.”
Consolidation	1	“I enjoyed the ILD TBL and feel it was very helpful in solidifying the information.”
Scaffolding	1	“It was helpful, especially when Dr. M gave us real-life experience on what to see and when.”
Areas for improvement (n = 14)		
Preference for lecture	6	“CBL takes a topic that could be taught in an hour to take three hours.”
Quality of preparation material	4	“Too many brand new concepts we were expected to master on our own with resources which were not thorough enough. Left me very confused.”
Organization	2	“Felt unprepared. I was unable to take notes during the lecture, which further inhibited my learning. Overall, I recall feeling frustrated.”
Scope and emphasis	2	“Very far out of scope.”

## Discussion

In agreement with prior data on the effectiveness of CBL [7-9,12,15] in enhancing student performance [7], students participating in our TB-CBL session scored higher on ILD-related final exam questions compared to those participating in didactic teaching. This observed performance improvement may be due to the collaborative nature of CBL, which facilitates peer-to-peer learning through active engagement, a structured approach to clinical problem-solving, guidance from experienced facilitators, and the promotion of positive team dynamics [12].

While we observed improved performance on ILD-related final exam questions (n = 6), it is important to acknowledge the small number of relevant MCQs on the final exam as a limitation of this study. In addition, Question 6 emerges as an outlier, with Cohort A (lecture) outperforming Cohort B (TB-CBL), possibly due to a greater emphasis on the temporal evolution of pathologic findings provided through the lecture. Finally, because a difference in overall final exam performance was identified between the cohorts, we cannot rule out the possibility that the observed improvement in performance on ILD-related questions may be due to baseline differences in cohort abilities or to the amount of classroom time dedicated to the topic (2.5 hours for Cohort A vs. 3 hours for Cohort B), rather than to the change in pedagogical approach. Moreover, it is important to note that our study assesses only short-term knowledge acquisition and satisfaction outcomes, not long-term retention or effects on clinical practice.

Student satisfaction with the session was acceptable but lower than expected, suggesting room for improvement in the preparatory material and organization of the session. Student comments also revealed that while many students felt that the session contributed to their learning, many others simply preferred lectures to active learning modalities. Modifications to the session may include the assignment of additional preparatory material and/or consideration of nonmandatory attendance.

A notable advantage of this activity is its adaptability to diverse learning environments and the specific educational needs of various programs and learners. In addition to modifications to the preparatory material, the in-class session may be shortened based on available timing and/or course-level learning objectives; it may be implemented in a large- or small-group setting; and it may be delivered in person or via remote virtual platforms.

Continued exploration of TB-CBL in medical education holds significant potential for enriching student learning experiences and improving outcomes [12]. By addressing the identified limitations and refining session design based on student feedback and educational best practices, educators can foster deeper understanding, critical thinking skills, and collaborative competencies among future healthcare professionals [11,12]. Additionally, ongoing research efforts focused on long-term retention and the translation of learning into clinical practice will provide valuable insights into the enduring impact of TB-CBL on medical education and patient care [12].

## Conclusions

We describe here a unique active learning session that may be adapted by medical educators at other institutions aiming to utilize active learning methods to teach the principles of diagnosis and management of ILD to preclinical medical students. Understanding ILD is paramount for medical students due to its profound global health impact. By equipping future healthcare professionals with the knowledge and skills to recognize, diagnose, and manage ILD effectively, we may be able to improve patient outcomes, alleviate suffering, and reduce the burden of this condition on public health systems.
